# A narrative review on current duodenoscope reprocessing techniques and novel developments

**DOI:** 10.1186/s13756-021-01037-z

**Published:** 2021-12-23

**Authors:** Maarten Heuvelmans, Herman F. Wunderink, Henny C. van der Mei, Jan F. Monkelbaan

**Affiliations:** 1 Department of Medical Microbiology, University Medical Center Utrecht, G04.643, PO box 85500, 3508GA Utrecht, The Netherlands; 2 Department of Biomedical Engineering, University of Groningen and University Medical Center Groningen, Groningen, The Netherlands; 3 Department of Gastroenterology and Hepatology, University Medical Center Utrecht, Utrecht, The Netherlands

**Keywords:** Endoscopy, Reprocessing, Infection, Outbreak, Multidrug-resistant Enterobacterales

## Abstract

Duodenoscopy-associated infections occur worldwide despite strict adherence to reprocessing standards. The exact scope of the problem remains unknown because a standardized sampling protocol and uniform sampling techniques are lacking. The currently available multi-society protocol for microbial culturing by the Centers for Disease Control and Prevention, the United States Food and Drug Administration (FDA) and the American Society for Microbiology, published in 2018 is too laborious for broad clinical implementation. A more practical sampling protocol would result in increased accessibility and widespread implementation. This will aid to reduce the prevalence of duodenoscope contamination. To reduce the risk of duodenoscopy-associated pathogen transmission the FDA advised four supplemental reprocessing measures. These measures include double high-level disinfection, microbiological culturing and quarantine, ethylene oxide gas sterilization and liquid chemical sterilization. When the supplemental measures were advised in 2015 data evaluating their efficacy were sparse. Over the past five years data regarding the supplemental measures have become available that place the efficacy of the supplemental measures into context. As expected the advised supplemental measures have resulted in increased costs and reprocessing time. Unfortunately, it has also become clear that the efficacy of the supplemental measures falls short and that duodenoscope contamination remains a problem. There is a lot of research into new reprocessing methods and technical applications trying to solve the problem of duodenoscope contamination. Several promising developments such as single-use duodenoscopes, electrolyzed acidic water, and vaporized hydrogen peroxide plasma are already applied in a clinical setting.

## Introduction

Duodenoscopes are diagnostic and therapeutic instruments used to visualize the interior of the upper gastrointestinal tract, collect tissue samples and perform therapeutic interventions. Inherent to their use duodenoscopes carry the risk of acquiring microbial contamination [1]. Over the past decade duodenoscopy-associated outbreaks often caused by multidrug-resistant (MDR) bacteria have been reported worldwide [2–15]. A report of the Emergency Care Research Institute in 2020 included sterile processing errors in medical and dental offices in the top ten of health technology hazards reflecting the increasing awareness and recognition of the risks of endoscopy-associated pathogen transmission in general [16]. Duodenoscopy-associated infections and outbreaks have also occurred despite strict adherence to reprocessing standards [4, 6, 13, 15].

The most common organisms involved in duodenoscopy-associated transmission are *Klebsiella pneumoniae* and *Pseudomonas aeruginosa* [2–15]. These bacterial pathogens are known for their biofilm formation and likelihood of multidrug resistance [11, 17, 18]. The predominance of MDR bacteria in duodenoscopy-associated outbreaks is most likely related to the fact that duodenoscopy-associated outbreaks are mainly noticed as a result of an elevated incidence of a specific MDR bacterium demanding infection control measures [2–15]. An elevated incidence of non-MDR bacteria is more likely to go unnoticed, leading to underestimation of duodenoscopy-associated pathogen transmission [8–10]. Duodenoscope contamination rates after reprocessing vary between 0.4 and 35.8%. This high variability can be explained by the absence of standardized sampling and culture methods and different definitions used for duodenoscope contamination (Table 1) [19–30]. Duodenoscope contamination is defined as growth of a specific predefined set of oral and/or gastrointestinal bacteria or as growth above a predefined threshold of any type of microorganism regardless of origin. This is for example described in the professional standard of the Dutch steering group for flexible endoscope cleaning and disinfection and the guideline of the European Society of Gastrointestinal Endoscopy [31, 32]. Although such guidelines have clear definitions, in literature a wide array of definitions has been used (Table 1). This illustrates the lack of a uniform and clear definition, and a standardized protocol [19–30].

**Table 1:** Culture positivity rate of duodenoscopes after strict adherence to reprocessing standards

First definition of contamination	CPD (%)	Second definition of contamination	CPD (%)	References
Any growth of high-concern organisms^a^ or > 10 CFU of low-concern organisms^b^	18%	NA	NA	[29]
Any growth of high-concern organisms^c^	4.9–5%	> 100 CFU of low/moderate concern organisms^d^	0.6–4.4%	[21]
Any growth of high-concern organisms^e^	0.4%	Growth of any organism	7.7%	[30]
Growth of any organism	1.1%	NA	NA	[26]
Micro-organism of gastro-intestinal or oral origin regardless of quantity^f^	15%	Growth of any organism ≥ 20 CFU/ml	22%	[19]
Any growth of Gram-negative bacilli^CDC^	4.2%	NA	NA	[25]
Growth ≥ 10 CFU/ml on the elevator mechanism or working channel	2.3%	Growth of any organism on the elevator mechanism or working channel	16.1%	[28]
Any growth of pathogenic organisms^g^	0.9%	Growth of any organism	8.4%	[23]
≥ 50 CFU/ml excluding skin contaminants^h^	0.9%	Growth of any organism	11%^i^	[20]
Any growth of high-concern organisms^CDC^	2%	Growth of any organism^CDC^	13.1%	[22]
> 100 CFU of total growth or any growth of high-concern organisms^j^	35.8%	Growth of any organism above ≥ 25 CFU	NA	[27]

Only studies published in the last decade have been included. Most studies report two definitions of contamination with accompanying contamination rates

*CPD* culture positive duodenoscopes, percentage of duodenoscopes regarded contaminated according to the definition given; *CFU/ml* number of colony forming units per milliliter; *NA* not applicable; ^*CDC*^ sampling was performed using the centers for disease control and prevention interim sampling protocol released in 2015

^a^Yeast, *Staphylococcus aureus*, *Enterococcus* species, Gram-negative enteric bacilli

^b^coagulase-negative *Staphylococcus* species, *Micrococcus* species, Gram-positive rods

^c^Gram-negative rods, *Staphylococcus aureus,* beta-hemolytic *Streptococcus, Enterococcus* species, Yeast

^d^Undefined

^e^*Escherichia coli*, *Enterococcus faecalis*, *Enterococcus faecium*, *Enterococcus* species, *Enterobacter cloacae*, *Aeromonas* species

^f^Yeast, *Klebsiella* species, *Escherichia* species, *Enterobacter* species, *Enterococcus* species, *Pseudomonas aeruginosa*, *Staphylococcus aureus*, *Moraxella* species, *Rothia* species, *Streptococcus* species, and *Neisseria* species

^g^Enteric Gram-negative bacilli, *Pseudomonas aeruginosa*, *Acinetobacter baumannii*, *Staphylococcus aureus*, *Enterococcus* species and *Stenotrophomonas maltophilia*

^h^Organisms not defined

^i^No number per endoscope provided, only a number of contaminated samples is available

^j^*Staphylococcus aureus*, Enterobacterales, *Pseudomonas* species, *Stenotrophomonas maltophilia*, *Acinetobacter* species and *Candida* species

This narrative review aims to give an overview of the problems associated with duodenoscope reprocessing. Novel promising developments for reprocessing of duodenoscopes are summarized and discussed. The literature search for this narrative review used the search term “endoscop*” which was combined with the different search terms regarding the topics discussed in this review on PubMed. Articles were first screened for eligibility based on title and abstract and all remaining full text articles were screened completely. A second search was performed on PubMed for all topics without the term “endoscop*” to see if any articles were missed. Finally the selected articles were cross-referenced to reveal any additional missed articles.

## Duodenoscope reprocessing

**Fig. 1:**
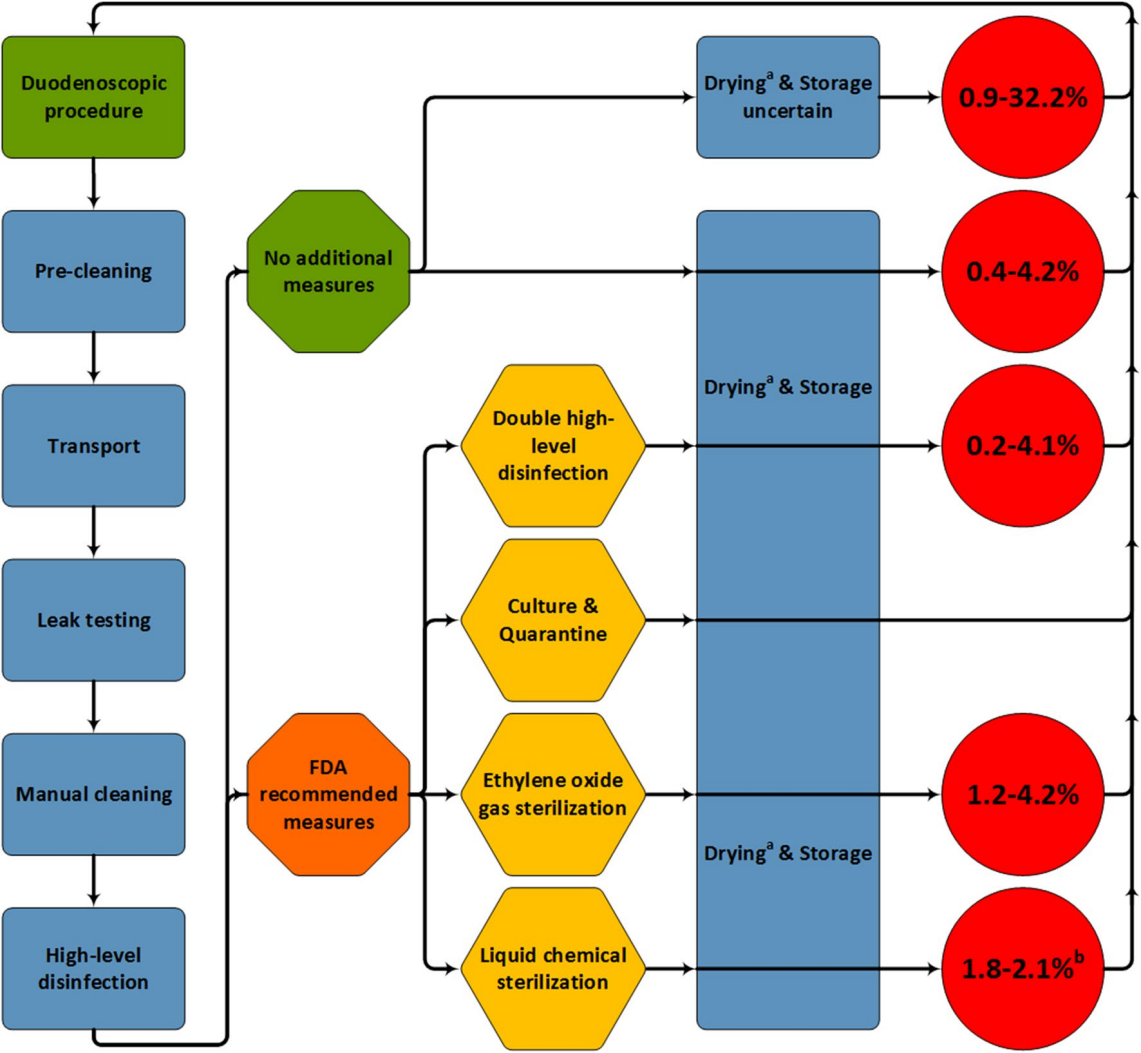
Duodenoscope contamination rates after standard duodenoscope reprocessing and the supplemental reprocessing measures. The green rectangle represents the duodenoscopic procedure and the blue rectangles show the current standard of reprocessing [33]. The green octagon represents the standard reprocessing procedure and the orange octagon represents the addition of one of the supplemental reprocessing measures to the standard reprocessing procedure. After standard reprocessing, several studies describe drying and storage and others did not. The four supplemental measures which were advised by the FDA in 2015 are depicted in yellow hexagons [57]. Red circles indicate contamination rates [19–30, 59, 80, 93]. ^a^ Some guidelines allow limited drying-time [38, 42]. ^b^ One study used double HLD containing peracetic acid and hydrogen peroxide [29]

The different guidelines used for duodenoscope reprocessing all use several comparable principal steps (Fig. 1) [33]. Variation within each step is common such as the chemical used for high-level disinfection (HLD) or the type of duodenoscope reprocessor. Immediately following the completion of the duodenoscopic procedure, reprocessing starts with a pre-cleaning step by wiping the outside of the duodenoscope with a cloth immersed in an enzymatic solution, flushing the channels with a detergent and removing all detachable parts of the duodenoscope [32–36]. After pre-cleaning the duodenoscope is transported to a cleaning facility where leak tests and manual cleaning are performed. Leak testing is performed to identify openings in the duodenoscope that can lead to entry of liquids, chemicals and organic debris [32–36]. Manual cleaning should be performed in accordance with manufacturer instructions and usually consists of submersion in a detergent solution, flushing and brushing of duodenoscope channels and cleaning of duodenoscope valves and the elevator mechanism. Directly afterwards the duodenoscope is thoroughly rinsed with water to remove any residual detergent and debris that has been dislodged during the manual cleaning [32–36]. After manual cleaning HLD is performed which involves rinsing and flushing of the duodenoscope with a chemical such as glutaraldehyde that is capable of killing all micro-organisms on the outside and inside of the duodenoscope except for bacterial spores [1]. HLD can be performed manually or with an automated endoscope reprocessor. The last reprocessing step consists of drying and storage preferably in a cabinet facilitating drying by forced air flowing through the cabinet or through the duodenoscope channels [37]. It has been shown that when a duodenoscope is re-used within 3–4 h after HLD, only ten minutes of drying-time is sufficient to prevent bacterial growth [32, 34, 38–44]. When duodenoscopes are stored longer than 3–4 h continued drying is essential for duodenoscope reprocessing as even limited amounts of residual moisture may promote bacterial growth and biofilm formation [37, 42–45].

## Reprocessing failure

The reprocessing procedure is essential because during use of duodenoscopes both the out- and inside are exposed to body fluids and potential contamination requiring disinfection before re-use of the duodenoscope [16]. This means that duodenoscopes go through a constant cycle of contamination and reprocessing. During these cycles organic material (extracellular matrix) and bacteria accumulate inside the duodenoscope channels also known as cyclic reprocessing build-up [46]. Deposition of organic material can further be facilitated by imperfections in the inner lining of the duodenoscope channels. These imperfections can already occur shortly after initial commissioning of the duodenoscope [47, 48]. Accumulation of cyclic reprocessing build-up and subsequent seeding of this cyclic reprocessing build-up with bacteria will eventually lead to bacterial biofilm formation [39, 47–52]. Another important factor complicating the reprocessing of duodenoscopes is their complex design which contributes to reprocessing failure [10–14, 53]. Design issues have been linked to reprocessing failure and even outbreaks in the past [10, 11]. The high duodenoscope contamination rate after reprocessing despite strict adherence to reprocessing protocols, strongly suggests that residual organic debris and bacterial biofilm formation occur (Table 1) [19–30, 52, 54–56].

The United States Food and Drug Administration (FDA) issued a statement in 2015 advising supplemental reprocessing measures for all duodenoscopes to minimize reprocessing failures. These supplemental reprocessing measures included double HLD, microbiological culturing and quarantine, ethylene oxide (EtO) gas sterilization or use of a liquid chemical sterilant [57]. By 2016, 89.6% of surveyed healthcare facilities in the United States of America implemented at least one of these measures, with double HLD (63%) and microbiological culturing and quarantine (53%) used most frequently [58].

## Double high-level disinfection

Double HLD consists of either a second cycle of HLD or repetition of the entire reprocessing procedure [28–30, 59]. This dual definition of double HLD and the fact that different studies use different HLD chemicals makes comparison between studies difficult [28–30, 59]. The additional time added to the entire reprocessing procedure by a second cycle of HLD is less than one hour and the additional costs are only $68.55 resulting in the lowest added costs of all the supplemental measures [60].

To date both approaches of double HLD have shown no reduction of the contamination rate of duodenoscopes [28, 30, 59]. A reduction in contamination rate has been shown after several subsequent cycles of peracetic acid based HLD, the contamination rate decreased from 18% after the first cycle to below 1% after the third cycle [29]. Thus, although the increased reprocessing time and additional costs incurred by double HLD are limited, most data suggest that a second cycle of HLD is not effective.

## Microbiological culturing and quarantine

Microbiological culturing and quarantine means that all duodenoscopes are sampled after reprocessing for microorganism detection. This entails that all duodenoscopes need to be quarantined until they are confirmed culture negative. When pathogens are identified from a reprocessed duodenoscope the duodenoscope needs to be reprocessed, cultured and quarantined again. Once cultures are definitively negative another reprocessing cycle should be performed before releasing the duodenoscope for clinical procedures. Persistently contaminated duodenoscopes should be evaluated by the manufacturer for internal damage and in case of MDR bacteria, patients exposed to the contaminated duodenoscope involved should be notified and considered for screening for their MDR bacterial carrier status [24].

Implementation of microbiological culturing and quarantine is costly and time consuming as it requires a microbiological infrastructure, trained personnel, enough duodenoscope storage capacity and a large number of duodenoscopes to continue duodenoscopic procedures while awaiting culturing results of reprocessed duodenoscopes that are quarantined [44, 60, 61]. However, in a non-outbreak setting periodical sampling of duodenoscopes is a more cost-effective option ensuring that all duodenoscopes are cultured over a preset period of time. Furthermore, in such a setting it will probably be worthwhile not to quarantine duodenoscopes while awaiting culture results [14, 24, 26, 62]. Of course when a duodenoscope is not quarantined pending culture results a positive microbiological culture will demand surveillance of patients exposed and outbreak control management.

There are different methods for duodenoscope sampling which vary in their sensitivity for detecting microbiological contamination (Table 2) [61, 63–68]. This variability of sensitivity possibly explains why outbreaks with a clear epidemiological link to a duodenoscope have revealed no contamination of the duodenoscope when cultures were taken [5, 6, 14]. Standardized methods for duodenoscope sampling and a uniform definition of duodenoscope contamination are lacking and current definitions are based on growth of specific pathogens, number of colony forming units or both (Table 1) [19–30]. This is the reason that reported duodenoscope contamination rates after reprocessing range from 0.4 to 35.8% (Table 1) [19–30].

**Table 2:** Duodenoscope sampling methods

Method	Principle	Current status	Advantages	Disadvantages	References
Flush	Release of bacteria through fluid flush	Clinically applied	Least complex sampling technique	Obsolete due to increased recovery with flush brush flush protocols	[64, 66, 68]
Flush Brush flush (CDC, FDA, ASM protocol)*	Release of bacteria through dual fluid flush and mechanical removal	Clinically applied	Can be considered as the current standard method of endoscope sampling	Requires two persons for sampling and is too laborious for general implementation	[61, 65, 67]
Flush brush flush (other protocols)	Release of bacteria through dual fluid flush and mechanical removal, uses different brushes and/or flushing fluid	Only applied in experimental models	The CDC, FDA and ASM protocol is outperformed in regard of Gram-negative bacteria recovered	Not tested in a clinical setting and a uniform protocol is still lacking	[63–65]
Pump assisted	Uses a peristaltic pump to increase shear stress at the lumen surface to remove bacteria	Clinically applied	More bacterial recovery compared to the flush method	Requires a peristaltic pump	[66]
Turbulent fluid flow	Adds turbulent air droplets to the flushing fluid to achieve high shear stress at the lumen surface to remove bacteria	Only applied in experimental models	More bacterial recovery compared to the flush method and the CDC flush brush flush method	Requires a device to generate turbulent flow	[67]
Tensioactive agents	A tensioactive agents is added to decrease surface tension of the sampling fluid thus aiding bacterial removal	Only applied in experimental models	Only requires addition of a tensioactive agent to the sampling fluid	Limited data are available and have reported conflicting results in regards of efficacy	[63, 68]

*CDC Centers for Disease Control and Prevention, *FDA* United States Food and Drug Administration, *ASM* American Society for Microbiology

The only multi-society protocol available for microbial culturing was released in 2018 by the Centers for Disease Control and Prevention (CDC), the FDA and the American Society for Microbiology (ASM) [61]. This protocol is divided in two sections describing the microbiological sampling and culturing methods. Microorganisms are classified as low- moderate- and high-concern and the protocol defines which actions are required in case of a positive microbiological culture. Gastrointestinal microorganisms such as Enterobacterales and *Pseudomonas aeruginosa* are always regarded as high-concern regardless of quantity. Microbiological flora such as coagulase-negative *Staphylococcus* species and *Micrococcus* species are defined as low/moderate-concern organisms and action is only required when the number of colony-forming units (CFU/ml) exceeds 100 CFU/ml. Given that this protocol contains more than 100 steps and requires two persons for sampling makes it too laborious for use in clinical practice and general laboratories [61].

In conclusion, the high costs associated with a complete microbiological culturing and quarantine program preclude this measure as a definite solution for duodenoscope-associated transmission. However, microbiological culturing and quarantine protocols have shown to be useful in identifying failures in reprocessing procedures [24, 69, 70].

## Ethylene oxide gas sterilization

EtO has potent alkylating properties resulting in sterilization and has been used for at least 40 years [71–73]. EtO allows sterilization of instruments with thermolabile materials such as duodenoscopes due to the low temperature typically 50 °C at which the sterilization can be performed compared to the higher temperatures needed with other sterilization methods. In the past mixtures containing EtO with chlorofluorocarbon, hydrochlorofluorocarbons or carbon dioxide have been used. However, nowadays they are replaced by 100% EtO gas due to environmental issues [74–76]. EtO is flammable and has carcinogenic properties and should therefore be handled with care [77].

In the presence of organic deposition and/or biofilm the efficacy of EtO is reduced due to limited penetration in organic materials and in a clinical setting no added benefit has been found from adding EtO gas sterilization after HLD [28, 75, 78, 79]. Another disadvantage of EtO gas sterilization is that it is time-consuming. It takes approximately 13 h, one hour exposure time with a 12-h aeration cycle due to absorption of EtO in the polymer materials of the duodenoscope creating a high burden on duodenoscope availability and decreasing cost-effectiveness [60, 80–82].

## Liquid chemical sterilization

In contrast to an agent suited for HLD a sterilizing agent such as peracetic acid, sodium hypochlorite or hydrogen peroxide can also effectively kill bacterial spores [83, 84]. The strong oxidizing properties of these chemicals leads to sterilization of the device. Unfortunately, this strong oxidizing effect also results in corrosion of parts in the duodenoscopes which is why sodium hypochlorite and high concentrations of hydrogen peroxide are not used in clinical practice [85, 86].

Peracetic acid (1820 mg/l) is effective in sterilizing duodenoscopes of different manufacturers [87]. Effectivity has also been shown in colonoscopes contaminated with *Enterococcus faecalis* and in bronchoscopes contaminated with *Mycobacterium gordonae* [82, 88, 89]. Concerning organic deposition peracetic acid is superior to EtO gas because it removes organic deposition through flow [75]. The efficacy of peracetic acid against bacteria in biofilm is comparable to *O*-phatalaldehyde and is superior to glutaraldehyde [90]. It should however be noted that certain peracetic acid formulations can have a fixating effect on biofilm [91, 92]. In a clinical setting peracetic acid has been compared to double HLD revealing similar effectiveness [93]. Peracetic acid has also been used as HLD chemical but revealed contamination rates comparable to other HLD chemicals [29]. It should also be noted that peracetic acid is a highly toxic chemical and requires handling precautions [94, 95]. Current data suggest that contamination of duodenoscopes still occurs after liquid chemical sterilization. Implementation of liquid chemical sterilization with peracetic acid requires limited modifications to the current reprocessing procedure because it only needs replacement of the chemical used in the HLD step. Therefore additional costs and added time will be limited compared to other interventions, such as EtO.

## Future perspectives

Despite the disappointing results of the supplemental measures several promising innovations are under development such as, single-use duodenoscopes, bioburden assays, electrolyzed acidic water, vaporized hydrogen peroxide plasma, cavitation, methylene blue photodynamic therapy and plasma-activated gas (Table 3) [73, 79, 96–118]. Unfortunately, the lack of a uniform protocol and definition makes it difficult to simulate duodenoscope reprocessing in experimental models. Therefore, evaluation of the effects of these new methods and their usefulness compared to current reprocessing standards is difficult.

**Table 3:** New methods for duodenoscope reprocessing

Method	Phase of development	Advantages	Disadvantages	References
Single-use duodenoscope	Implemented	No need for reprocessing, non-toxic	High costs, quality of duodenoscope	[96–98]
Bioburden assays	Implemented	Quick and easy to use	Lack of correlation with microbial culture	[99–103]
Electrolyzed acidic water	Endoscope tested	No biofilm fixation, non-toxic	Preparation on site needed	[104–110]
Vaporized hydrogen peroxide plasma	Endoscope tested	No aeration needed, non-toxic	Material incompatibility	[73, 79, 111–115]
Cavitation	Not tested	Potentially effective against biofilm, non-toxic	No disinfecting properties	[116]
Methylene blue photodynamic therapy	Model tested	Effective against biofilm, limited toxicity	Practical application lacking	[117]
Plasma-activated gas	Model tested	Non-toxic	Short-lived effect	[118]

### Single-use duodenoscopes

In 2019, the FDA approved the first single-use duodenoscopes [96]. Their use completely obviates the need for reprocessing but incurs significant higher costs compared to reusable duodenoscopes [97]. Cost-effectiveness will therefore depend on multiple factors such as rate of reusable duodenoscopy-associated infections, costs related to a possible duodenoscopy-associated infection or outbreak, the number of procedures performed annually and the performance of these single-use duodenoscopes compared to reusable duodenoscopes [97, 98]. Furthermore single-use duodenoscopes will without doubt lead to more waste and have an increased environmental impact.

### Bioburden assays

Bioburden assays are rapid and low-cost tests which can detect the presence of organic soil by detecting biomarkers such as protein, hemoglobin or adenosine triphosphate (ATP) [99, 101]. Benchmarks regarding detection of protein or ATP have been described and used to evaluate the efficacy of manual cleaning [99–101]. ATP has also been used to evaluate duodenoscope contamination but presence of ATP in organic material other than viable bacteria causes a poor correlation between ATP levels and bacterial contamination [101–103]. Furthermore, it should be noted that the sensitivity of bioburden assays will depend on the sampling method used. Given that a standardized method for collection of samples for testing with bioburden assays in duodenoscopes is currently unavailable precludes broad implementation.

### Electrolyzed acidic water

To prepare electrolyzed acidic water an electric current is run through a saline solution resulting in acidity, hypochlorite ions and free chlorine which all contribute to bactericidal activity [104]. Efficacy has been shown in both a contaminated gastrointestinal endoscope model and clinical practice, and no bacteria were recovered when electrolyzed acidic water treatment was used for at least 5 min [104, 105]. The efficacy of electrolyzed acidic water depends on the amount of organic debris because of uptake of the bactericidal chemicals in the organic debris therefore adequate manual cleaning prior to use is warranted [106]. Interestingly, in a model utilizing metal cylinders without organic debris some cylinders still harbored viable bacteria after electrolyzed acidic water treatment [107]. Electrolyzed acidic water has been compared to glutaraldehyde and has comparable efficacy based on contamination rate [106, 108–110]. The advantages of electrolyzed acidic water are that it does not leave toxic residues, does not fixate proteins and thereby is less likely to promote biofilm [107]. A disadvantage of electrolyzed acidic water is that it needs to be used immediately after preparation because the efficacy of the solution decreases rapidly due to the unstable nature of the ions formed [106]. This complicates the reprocessing procedure because efficacy of HLD chemicals needs to be guaranteed through testing prior to use. Therefore if electrolyzed acid water is implemented this will increase reprocessing time because both preparation and testing of the solution need to be performed shortly before reprocessing of each duodenoscope. Electrolyzed acidic water has been cleared by the FDA since 2002 as a high-level disinfectant.

### Vaporized hydrogen peroxide plasma

In this method hydrogen peroxide vapor is used as primary sterilizer which in a second step is stimulated to form plasma and therewith antimicrobial free radicals such as hydroxyl and hydroperoxyl. The process results in low temperature sterilization without toxic byproducts and does not require an aeration cycle such as EtO [73, 111]. Vaporized hydrogen peroxide plasma in laboratory studies has been shown to have potent sterilizing activity in long narrow lumens however when serum and salts are present efficacy is reduced with 65% due to hydrogen peroxide reacting with serum and salts [73, 79, 111]. No contamination was observed when experimentally contaminated flexible gastrointestinal endoscopes with *Geobacillus stearothermophilus* spores were examined for residual contamination after application of vaporized hydrogen peroxide plasma [111–114]. Vaporized hydrogen peroxide has also been used in conjunction with ozone as an additional sterilant which resulted in complete sterilization of duodenoscopes [115]. Vaporized hydrogen peroxide plasma sterilizers are a promising development for reprocessing, however data regarding clinical application are still limited. Furthermore there is still a matter of material incompatibility because the molybdenum disulphide lubricant used in duodenoscopes reacts with hydrogen peroxide creating corrosive acids that disintegrate the epoxy resin of the duodenoscope [114]. These material incompatibility issues will need to be solved before FDA approval or widespread implementation is considered.

### Cavitation

Cavitation is a phenomenon in fluid dynamics where spherical cavities (microbubbles) are generated in a fluid through ultrasound [116]. Microbubbles interact with biofilm through a cycle of microbubble generation and collapse. Collapsing of these bubbles close to bacteria leads to bacterial damage and biofilm disintegration. Although cavitation could be a promising method for removal of biofilm during reprocessing it does not have disinfecting properties. Therefore cavitation can potentially be used prior to HLD for removal of biofilm and possibly increase the effectiveness of disinfection. Further research will be needed to determine if such strategies are feasible.

### Methylene blue photodynamic therapy

This method uses laser light to induce reactive oxygen species in a methylene blue solution that exerts a bactericidal effect. The method can be enhanced by adding hydrogen peroxide which will also make it effective in the presence of biofilm [117]. Until now this method has only been used in an experimental model. The short half-life of reactive oxygen species makes it difficult to apply this method to duodenoscopes.

### Plasma-activated gas

This method uses an electrical current to induce a plasma state in argon gas. This plasma-activated gas is directed through the duodenoscope channel to induce bactericidal reactive oxygen and nitrogen species [118]. Given that plasma-activated gas only produces short-lived (microseconds) reactive oxygen and nitrogen species, its toxicity is limited and therefore does not require an aeration cycle like EtO [118]. Currently this technique has only been applied in a polytetrafluoroethylene test tube model and therefore further research will be necessary to evaluate if plasma-activated gas is also effective and practical in a clinical setting.

## Discussion

Duodenoscopy-associated infections and outbreaks occur worldwide but the exact scope of the problem is unknown [2–15]. Reported outbreaks almost exclusively involve MDR bacteria making it likely that outbreaks with other (non-MDR) microorganisms remain undetected [2–15]. Monitoring and estimation of duodenoscopy-associated transmission can be achieved by determining the contamination rate. The only available guideline for sampling and culturing of duodenoscopes is a multi-society guideline of the CDC, the FDA and the ASM [61]. Unfortunately, this guideline contains over 100 steps making it too laborious for widespread implementation in general laboratories [61]. Therefore development of a uniform and foremost more practical sampling protocol for duodenoscope contamination is of the utmost importance because this would results in more widespread implementation. New sampling methods for microbiological culturing can play a major role in such a protocol in the future (Table 2) [63–67, 70]. If such a uniform and more practical sampling protocol for duodenoscope contamination is developed this will facilitate data collection. When more data regarding duodenoscope contamination become available this will lead, to not only a more precise estimation of the duodenoscope contamination rate, but will also facilitate collection in a central database and lead to opportunities to examine new reprocessing techniques and procedures.

The implementation of the FDA proposed supplemental reprocessing measures to improve duodenoscope reprocessing outcomes has not resulted in reduction of the duodenoscope contamination rate (Fig. 1) [19–30, 57, 59, 80, 93]. Implementation of the supplemental measures has however increased reprocessing time and costs. Therefore continued use of the supplemental measures to reduce the duodenoscope contamination rate is not sensible.

Perhaps the solution to the problem of ongoing bacterial contamination of duodenoscopes after reprocessing can be found in novel technical applications and reprocessing techniques. Newly proposed methods for improvement of duodenoscope reprocessing are promising and some have already been applied in the reprocessing procedure of duodenoscopes (Table 3) [73, 79, 96–118]. Several other promising methods however still remain in a very preliminary phase of development and will benefit from a practical standardized sampling protocol so their effect on the duodenoscope contamination rate can more easily and rapidly be determined (Table 3) [116–118]. This would surely aid in determining their usefulness in the reprocessing procedure and allow comparison to current reprocessing techniques and procedures.

Single-use duodenoscopes would surpass the current problems of reprocessing. However, these single-use duodenoscopes are expensive, increase waste and cost-effectiveness will depend on performance, rate of infection, costs incurred per infection and number of procedures performed annually [96–98]. Bioburden assays can contribute to monitoring of manual cleaning however they are unsuited to replace microbiological culturing [99–103]. Furthermore lack of a uniform and standardized protocol for sampling of duodenoscopes precludes implementation of bioburden assays.

Implementation of novel reprocessing methods that will replace parts of or add-on to the existing reprocessing methods such as electrolyzed acidic water, vaporized hydrogen peroxide plasma, cavitation methylene blue photodynamic therapy or plasma-activated gas suffer from lack of a uniform and practical sampling protocol. Lack of such a duodenoscope sampling protocol makes comparison to current reprocessing methods and techniques difficult.

## Conclusions

Reported duodenoscope contamination rates show that the current reprocessing methods are inadequate and underline the need for further research into novel reprocessing methods to improve reprocessing results so that duodenoscopy-associated infections and outbreaks can be prevented in the future. Development of a uniform and practical protocol for duodenoscope sampling that can be applied in general healthcare facilities is urgently needed and will facilitate development of novel reprocessing techniques because comparison between current and novel reprocessing methods can be more readily made.

## Data Availability

Not applicable.

## Abbreviations

MDR: Multidrug-resistant
HLD: High-level disinfection
FDA: United States Food and Drug Administration
EtO: Ethylene oxide
CDC: Centers for Disease Control and Prevention
ASM: American Society for Microbiology
CFU/ml: Colony-forming units per milliliter
ATP: Adenosine triphosphate
CPD: Culture positive duodenoscopes
NA: Not applicable
